# Extremism, knowledge, and overconfidence in the covid-19 restriction times

**DOI:** 10.3389/fpsyg.2024.1295807

**Published:** 2024-02-01

**Authors:** Tsuyoshi Hatori, Netra Prakash Bhandary

**Affiliations:** Department of Environmental Design, Ehime University, Matsuyama, Japan

**Keywords:** extremism, COVID-19 restrictions, knowledge, illusion of understanding, mechanistic explanation task

## Abstract

Public response to restriction policy against the novel coronavirus disease (COVID-19) can polarize into two extremes: one absolutely in favor of restrictions for the sake of human life and health, and other absolutely against the restrictions for the sake of human rights and daily life. This study examines psychological nature of extremism regarding individuals’ self-restraint from social behavior, which was and has been encouraged by the Japanese government as restriction measures, as well as possible measures to mitigate this extremism. We hypothesize that people with more extreme views on self-restraint tend to have less knowledge of this virus, and, nevertheless, tend to be more overconfident in the sense that they falsely believe they understand COVID-19 and the effects of self-restraint. It is also postulated that overconfidence can be reduced by asking them to explain how self-restraint works. To test these hypotheses, we conducted an online experiment on the Japanese adults (*n* = 500) to measure the extent of their knowledge of COVID-19 and to examine the effect of explanation task on their understanding regarding COVID-19 and extremism. The results indicate that the extreme attitudes were associated with insufficient knowledge about the symptoms, risks, and characteristics of COVID-19. Moreover, their extreme attitudes tended to moderate through this experimental study to an extent that they realized they did not understand COVID-19 including the effects of self-restraint. This suggests that people with extremism may have been overconfident in their own understanding of the COVID-19 restrictions.

## 1 Introduction

While the novel coronavirus disease (COVID-19) posed and is still posing a significant risk to human life, restrictive policies including travel restrictions, non-essential business closures, school closures, mandatory masks, and social distancing rules to mitigate the risk have entailed significant economic losses and violations of civic rights in some extent (Jørgensen et al., 2021). Many studies have noted that during this contentious situation, public response to restriction measures has polarized into two extremes, creating serious conflicts in society (e.g., Dyer, 2020; Hart et al., 2020; Mehrotra, 2020; Kerr et al., 2021). Extreme attitudes on the pro side, termed as *Pro-extremism* in this study, seek thorough restrictions to suppress the spread of COVID-19 and sometimes show strong hostility towards those who do not comply with the restriction requests (Takahashi and Tanaka, 2020) while the extreme attitudes on the contrarian side, termed as *Con-extremism*, show a vocal opposition against the restrictions, as seen in media reports of anti-mask protests and COVID-19 conspiracies (Taylor and Asmundson, 2021; Jabkowski et al., 2023). Particularly in Japan, as a *Pro-extremism*, the phenomenon of ‘self-restraint police’ appeared during the state of emergency (Osaki, 2020), which is a colloquial term for ordinary citizens who harassed individuals or shops that did not comply with the government’s request to refrain from going out or opening for business. They also posted slanderous notices on the doors of restaurants that were open and damaged cars with out-of-prefecture license plates.

Unfortunately, however, both these extreme attitudes of people during emergencies need to be regarded as problematic while dealing with the public health crises. For instance, *Pro-extremism* may not successfully take into account the negative impacts of the restrictive measures, which the Congressional Budget Office (CBO) (2020) and Nicola et al. (2020) also report like social distancing and other restrictions may negatively affect the economy and may also increase the risk of social isolation and loneliness, which may further lead to increased risk of premature mortality and suicide (Tanaka and Okamoto, 2021; Ernst et al., 2022). Likewise, *Con-extremism* may reduce the effectiveness of restrictive policies by spreading resistance to compliance with the restrictions in society. This is problematic in light of a number of studies (e.g., Aravindakshan et al., 2020; Courtemanche et al., 2020; Damette and Huynh, 2023) that indicate that social distancing and wearing of mask can be effective in mitigating the spread of COVID-19. However, some critics also argued that the anti-masking movement actually victimizes some vulnerable groups such as BIPOC (i.e., Black Indigenous People of Color, People of Color), elderly, poor, and disabled people (Grunnawalt, 2021). If these extreme attitudes get even more polarized, the situation may lead to social fragmentation, bringing in further difficulties to make appropriate social decisions on public health policies to control the pandemic.

### 1.1 Psychological natures of extremism

This study empirically investigates the psychological natures of extremism regarding the COVID-19 restrictions with an aim of gaining insights into ways to mitigate the extreme attitudes. A problematic attitude found in extremism can be illustrated by a refusal to make trade-offs of one’s values with other values; such values are known as *protective values* (Baron and Spranca, 1997) or *sacred values* (Tetlock et al., 2000). On the one hand, those with *Pro-extremism* may refuse to make trade-offs of human life against other values (often economic values), but on the other, those with *Con-extremism* may deny the need for trade-offs of the value of economy and freedom with other values even if they are human life. Such people may get annoyed by a mere thought that the values they are upholding could be traded off against other values (Tetlock et al., 2000). Argument divide by the extreme positions makes it difficult for the health authorities to make trade-off efforts over the implementation of restrictive policies (Baron and Leshner, 2000).

So, in this study, we address two psychological factors underlying the development of extremism. One of the factors that potentially explain these extreme attitudes is lack of knowledge about COVID-19. People’s attitude towards COVID-19 and its control measures is affected by their knowledge about this disease (Zhong et al., 2020). Some of the past research work (e.g., Baron and Leshner, 2000; Tetlock et al., 2000), however, indicate that the attitude of rejecting value trade-offs results from unreflective overgeneralization of the meaning and consequences of such extreme positions. People may believe that no benefit would be sufficient to justify relieving restrictions without thinking much about possible benefits. Similarly, those who believe that restrictions are unnecessary may not have fully considered the effects or significance of those restrictions. We therefore conjectured that extreme attitude towards COVID-19 restrictions is associated with people’s limited knowledge about this disease.

The next question that strikes the mind is why those with the extremism absolutely support their strong opinions on restrictive measures even though they have only limited amount of knowledge on COVID-19. One possible answer to this question could be people with extreme views are overconfident that they better understand COVID-19 including the effects of its restrictive measures than they actually do. Overconfidence generally refers to a situation in which a person overestimates the accuracy of his or her knowledge (Fischhoff et al., 1977; Yates et al., 1996). This tendency is one of the so-called self-serving biases (Greenberg et al., 1982), in which people usually interpret facts to help maintain a positive image of themselves, and a high evaluation of one’s knowledge clearly contributes to a positive self-image (Thaller and Brudermann, 2020). This concept is closely related to the failure of metacognition, which refers to someone’s knowledge of his or her own knowledge (Kruger and Dunning, 1999). Although many studies report overconfidence on a variety of topics (e.g., Fischhoff et al., 1977; Yates et al., 1997), it is so far not well addressed in topics related to the COVID-19 restrictions.

Also, overconfidence can occur when a person falls into the *illusion of understanding* (Rozenblit and Keil, 2002) in a sense that the person falsely believes that he or she understands certain topics (Bailey, 2021; Meyers et al., 2023). Rozenblit and Keil (2002) demonstrate a phenomenon of illusion of understanding (explanatory depth) by experimentally showing that people tend to be overconfident in how well they understand everyday objects such as helicopters and pianos. In their experiment, they asked the subjects to explain the causal mechanisms by which these objects function and showed that people are prone to illusion of understanding as this experiment reduces people’s self-assessment of their own understanding.

Fernbach et al. (2013) applied Rozenblit and Keil’s experiment into the issue of political extremity and revealed that extreme political attitudes result from an illusion of understanding of the causal processes underlying policies. So, we speculate that extreme policy positions in the COVID-19 restriction times often relied on an overestimation of people’s understandings of the effectiveness of the restriction policy or COVID-19 itself as its target. If this speculation of ours is true, asking people to explain the mechanisms by which the restrictive policies have had effects on the society should highlight their lack of understanding and thus lead them to express more moderate political views.

### 1.2 The present study

**Figure 1:**
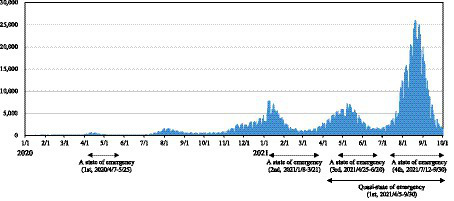
Number of positive cases of COVID-19 infection and declarations of a state of emergency. A state of emergency targets the entire prefecture, while a quasi-state of emergency targets some areas designated by the governor. Both declarations order or request businesses to refrain from activities, residents to refrain from outings, and events to be restricted, but there are differences in the degree of requests and penalties.

Based on the preceding discussion, in this study we address extremism associated with the restrictive measures in Japan and examine the knowledge of COVID-19 and the illusion of understanding. The Japanese government issued a state of emergency several times to prevent the spread of COVID-19, depending on the infection situation (Figure 1). Unlike in many other countries, Japan’s state of emergency is not legally binding; it is rather a request for self-restraint, and restriction measures against COVID-19 are left to individual judgment (Mori et al., 2022). When the Japanese government declares a state of emergency, citizens are required to voluntarily refrain from unnecessary and non-essential social activities including travel, eating, and drinking at a restaurant for extended periods of time and socializing with groups of five or more people (Cabinet and Secretariat, 2022). There has been recurring controversy over whether such self-restraint is acceptable (Borovoy, 2022). With this in mind, in this study, we focus on extremism regarding the self-restraint and posit the following two hypotheses:

*Hypothesis 1 (H1)*. Those with more extreme views on self-restraint tend to have less knowledge about the symptoms, risks, and characteristics of COVID-19.

*Hypothesis 2 (H2)*. Because those with extreme views falsely believe they have a better understandings of COVID-19 and the effects of self-restraint, their extreme attitudes tend to be mitigated to the extent that their self-rated understanding decline through the task of explaining the advantages and disadvantages of the self-restraint by themselves.

It should be noted here that the first hypothesis does not directly address knowledge of self-restraint. This is because at the time of this study, there was little true knowledge about the effects of self-restraint. Instead, this study addresses general knowledge (symptoms, risks, and characteristics) of COVID-19, which is also relevant to judging the validity of the restriction.

So, in this study, we tested the above two hypotheses on the Japanese adults (*n* = 500). We believe our findings contribute to further insights into extreme attitudes towards the COVID-19 restrictions. Most studies have analyzed public opinion towards the COVID-19 control measures with some of them also examining its relevance to knowledge of this disease (e.g., Jørgensen et al., 2021). However, only a few studies have focused on people’s extreme opinions and their psychological characteristics. We examine these extreme opinions and psychological characteristics, which we suppose may provide suggestions for measures to prevent extreme opinions about the risk of viral infections like COVID-19 and the polarization they cause.

## 2 Method

An online experiment, basically through a questionnaire was administered in conjunction with a market research company. The target of the experiment were five hundred Japanese adults who were recruited through the website using a sampling method with equal allocation in terms of gender and age; 50% male, 50% female, and 20% of each age group from 20s to 60s and above. The online survey was done, and the response data were collected in March 2021, which corresponds to a period between the third and fourth waves of infection in Japan, and it was the time when the number of infections started to increase (Figure 1).

**Figure 2:**
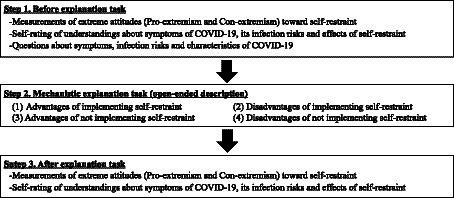
Experimental procedure.

The experimental procedure is shown in Figure 2. First, people’s extreme attitudes toward self-restraint from social behaviors were measured by asking the respondents the extent to which they absolutely agree or disagree with the self-restraint, rejecting the trade-offs associated with the self-restraint. They indicated their level of agreement with statements as listed in Table 1. We assumed the reduction of infection risk as the primary merit of self-restraint and the sacrifice of normal daily life, the Japanese economy, and the value of going out as their primary demerits. Then, for *Pro-extremism*, we asked respondents to rate how strongly they agreed with the self-restraint, prioritizing its merit value (infection risk), or sacrificing its demerit values (normal daily life, the Japanese economy, and the value of going out). Similarly, for *Con-extremism*, they were asked to rate the extent to which they strongly opposed the self-restraint with the demerit values (normal daily life, the Japanese economy, and the value of going out) as a top priority or at the expense of the merit value (risk of infection). All items were measured using a 7-point scale from 1 for *strongly disagree* to 7 for *strongly agree* with higher scores indicating greater extremism in favor of or against the restrictions. Measurements of *Pro-extremism* and *Con-extremism* were created by averaging across the respective 4 items and the satisfactory reliabilities were obtained (Pro-extremism: *α* = 0.91; Con-extremism: *α* = 0.93).

**Table 1:** Questionnaire items.

Pro-extremism	
	Priority of infection risk: *“Unless there is a zero chance of being infected with the COVID-19, I think we should thoroughly exercise self-restraint.”*
	Sacrifice of normal daily life: *“I think we should give priority to not spreading the infection anyway, no matter how much our normal lives are sacrificed.”*
	Sacrifice of Japanese economy: *“No matter how much the Japanese economy worsens, I think we should give priority to not spreading the infection anyway.”*
	Sacrifice of the value of going out: *“I think we should refrain from going out anyway until the infection of the COVID-19 is completely controlled.”*
Con-extremism	
	Sacrifice of infection risk: *“Even if there is a possibility of being infected with the COVID-19, I don't think it is necessary to restrain oneself at all.”*
	Priority of normal daily life: *“Even if there is a possibility of spreading the infection, I think that we should prioritize our normal life as before.”*
	Priority of Japanese economy: *“Even if there is a possibility of spreading the infection, I think priority should be given to promoting economic activities in Japan.”*
	Priority of the value of going out: *“Even if there is a possibility of being infected with the COVID-19, I don't think it is necessary to refrain from going out at all.”*
Self-rating of understanding	
	Symptoms of COVID-19: *“I think I fully understand the symptoms of the COVID-19.”*
	Infection risks of COVID-19: *“I think I fully understand the risk of infection by the COVID-19.”*
	Effects of self-restraint: *“I think I fully understand the effects and impacts of self-restraint from social and economic activities.”*

All items were measured using a 7-point scale from 1 for “*strongly disagree*” to 7 for “*strongly agree*”. As for question items about symptoms, infection risks, and characteristics of COVID-19, see the Supplemental material.

The respondents were also asked to rate their understanding of each of the COVID-19 symptoms, its risk of infection, and the effects and impacts of self-restraint. They indicated the extent to which they believe that they understand the symptoms of COVID-19 (or risk of infection; effects and impacts of self-restraint) well, using the same 7-point scale, with higher scores indicating greater understanding.

To measure their knowledge on COVID-19, several questions were asked in relation to its symptoms, infection risk, and its characteristics A total of 14 questions were asked on the symptoms (e.g., ‘Is malaise a symptom of COVID-19?’), 11 questions on the infection risk (e.g., ‘How likely do you think you are to become infected if you come in contact with an infected person?’), and 13 questions on the characteristics (e.g., ‘New coronaviruses are transmitted by asymptomatic carriers’). They were then asked how many questions they thought they answered correctly for each category. Their responses to the questions were evaluated for correctness based on the documents prepared by the WHO (2021) and Japan’s Ministry of Health, Labour and Welfare (2021). For the infection risk, an index of perceived risk was constructed by averaging across the participants’ responses to 11 questions.

Next, they were asked to provide a mechanistic explanation for self-restraint. They were asked to describe in an open-ended format the advantages and disadvantages of self-restraint from social and economic activities and non-restraint, respectively. This explanatory task, like the experiment conducted by Rozenblit and Keil (2002), is aimed at curbing the tendency towards the overconfidence, which were assessed through the self-ratings of their understanding regarding COVID-19 in the first session. To confirm this point, they were finally asked to re-rate their understanding of COVID-19 and their attitudes toward self-restraint in the same manner as in the first session.

## 3 Results

### 3.1 Descriptive analysis

The mean value for *Pro-extremism* was found to be higher than the median (4), in contrast to that for *Con-extremism* (Table 2). This suggests that the respondents were, as a whole, in favor of self-restraint; they tend to strongly support self-restraint, even at the expense of their daily lives, the national economy, and the opportunities to go out, unless the risk of infection by COVID-19 is reduced to zero. This preference for self-restraint was also found to be particularly greater among the women than among the men. These extreme attitudes remained generally high after the explanatory task and did not show any significant decrease compared to the pre-task levels. The mean self-rating of their understanding of COVID-19 was generally high, indicating that they tended to rate themselves as having a good understanding of COVID-19. Unlike previous studies such as by Rozenblit and Keil (2002), the reported understanding did not significantly decrease after the explanatory task. The rates of correct responses to the questions about COVID-19 remained low, around 60% at the best. In particular, those regarding the infection risk is only about 20%. However, the mean values of self-assessment for the correct response rates were all found to be higher than the actual rates, and the mean value for the infection risk is also close to 60%. The Pro-extremism is significantly positively related with the perceived risk (*r* = 0.32, *p* < 0.00), while the Con-extremism is significantly negatively related with it (*r* = −0.09, *p* < 0.05), which suggests that those with the pro-restriction extremism tend to be pessimistic about the risk of infection, while those with the anti-restriction extremism tend to be optimistic about it.

**Table 2:** Descriptive statistics.

			Gender		Age			Education		
	Before explanation	After explanation	Men	Women	20s-30s	40s-60s	60s-	Junior high school	High school	Collage
Pro-extremism [1–7]	4.37	4.27	4.27	4.46	4.50	4.17	4.51	3.11	4.55	4.30
	(1.35)	(1.37)	(1.51)	(1.17)	(1.36)	(1.37)	(1.27)	(2.27)	(1.23)	(1.38)
Con-extremism [1–7]	3.41	3.36	3.63	3.19	3.75	3.32	2.91	3.33	3.37	3.43
	(1.52)	(1.51)	(1.60)	(1.40)	(1.45)	(1.55)	(1.42)	(2.08)	(1.47)	(1.53)
Self-rating of understanding
Symptoms of COVID-19 [1–7]	4.53	4.78	4.53	4.53	4.51	4.61	4.42	5.67	4.42	4.56
	(1.20)	(1.03)	(1.26)	(1.14)	(1.30)	(1.11)	(1.19)	(2.31)	(1.20)	(1.19)
Infection risks of COVID-19 [1–7]	4.85	4.88	4.84	4.86	4.83	4.90	4.79	6.33	4.79	4.86
	(1.19)	(1.02)	(1.25)	(1.14)	(1.25)	(1.13)	(1.23)	(1.15)	(1.19)	(1.18)
Effects of self-restraint [1–7]	4.94	4.77	4.95	4.93	4.88	5.01	4.93	6.33	4.84	4.96
	(1.21)	(1.07)	(1.29)	(1.13)	(1.26)	(1.13)	(1.27)	(1.15)	(1.26)	(1.19)
Questions about symptoms of COVID-19
Percentage of correct answers [0–100]	53.27%	—	50.37%	56.17%	51.68%	54.21%	54.57%	35.71%	51.45%	54.49%
	(23.18%)		(23.88%)	(22.13%)	(22.72%)	(24.17%)	(22.07%)	(24.74%)	(24.81%)	(22.37%)
Self-assessment of percentage of correct answers [0–100]	68.10%	—	67.03%	69.17%	63.68%	70.36%	72.43%	69.05%	68.89%	68.06%
	(22.43%)		(24.37%)	(20.28%)	(22.75%)	(22.73%)	(19.66%)	(32.21%)	(22.98%)	(21.73%)
Questions about infection risks of COVID-19
Percentage of correct answers	22.65%	—	26.58%	18.73%	18.68%	25.27%	25.36%	36.36%	19.10%	23.63%
	(17.57%)		(19.28%)	(14.69%)	(16.49%)	(17.98%)	(17.49%)	(24.05%)	(16.81%)	(17.69%)
Self-assessment of percentage of correct answers [0–100]	57.13%	—	59.67%	54.58%	51.50%	59.05%	64.55%	60.61%	60.08%	56.18%
	(23.09%)		(24.34%)	(21.52%)	(21.95%)	(24.08%)	(20.65%)	(36.74%)	(23.47%)	(22.45%)
Questions about characteristics of COVID-19
Percentage of correct answers	62.09%	—	60.95%	63.23%	61.19%	63.35%	61.38%	58.97%	60.31%	62.67%
	(13.35%)		(14.18%)	(12.40%)	(13.91%)	(13.18%)	(12.47%)	(8.88%)	(12.20%)	(13.74%)
Self-assessment of percentage of correct answers [0–100]	62.12%	—	64.25%	60.00%	57.92%	63.88%	67.00%	61.54%	64.38%	61.33%
	(22.35%)		(23.11%)	(21.39%)	(22.65%)	(22.23%)	(20.64%)	(20.35%)	(21.80%)	(22.48%)

Numbers in the cells represent the mean and those in parentheses (•) represent the standard deviation. Numbers in brackets [•] represent the range of each scale.

### 3.2 Knowledge on COVID-19

To examine the amount of knowledge about COVID-19 among those with the extremism (H1), a regression analysis was done with *Pro-extremism* and *Con-extremism* as dependent variables and correct response rates and their self-assessment indicators as explanatory variables (Table 3). *Pro-extremism* was found to be significantly negatively associated with all correct response rates, indicating that the supporters of self-restraint tend to lack an overall knowledge about COVID-19. Nonetheless, this extreme attitude was found to be significantly positively associated with self-assessments of correct response rates on symptoms, indicating that they tend to rate their own correct response rates on symptoms higher than they actually did. Likewise, *Con-extremism* was found to be significantly negatively associated with correct response rates on symptoms and characteristics, indicating that the opposers of self-restraint tend to lack the knowledge of these. Self-assessment of correct response rate on symptoms has a significantly negative coefficient, which indicates that the respondents are, to some extent, aware of their low correct response rate. However, regarding the infection risk, it has a significantly positive coefficient, which indicates that they tend to evaluate their own correct response rate (with regard to the infection risk) higher than it actually is. Furthermore, *Con-extremism* showed a statistically significant negative association with the gender and age: the tendency is lower for women than men, and it decreases with increasing age.

**Table 3:** Results of regression analysis of extreme attitudes associated with knowledge-related variables.

	*Pro-extremism*		*Con-extremism*	
Explanatory Variables		*β*	t-stat.	*β*	t-stat.
Questions about symptoms of COVID-19	
Percentage of correct answers		−0.09*	−1.92	−0.12***	−2.68
Self-assessment of percentage of correct answers		0.11*	1.88	−0.12**	−2.01
Questions about infection risks of COVID-19	
Percentage of correct answers		−0.33***	−7.39	0.04	0.89
Self-assessment of percentage of correct answers		−0.03	−0.46	0.22***	3.57
Questions about characteristics of COVID-19	
Percentage of correct answers		−0.11***	−2.65	−0.12***	−2.85
Self-assessment of percentage of correct answers		0.05	0.79	−0.05	−0.89
Gender		0.01	0.26	−0.09**	−2.00
Age		0.01	0.18	−0.25***	−5.78
High school dummy		−0.03	−0.32	0.08	0.82
Collage dummy		−0.06	−0.65	0.10	0.99
Nagelkerke *R^2^*		0.12		0.12	

*p < 0.10.

**p < 0.05.

***p < 0.01.

Dependent variables of Pro-and Con-extremism were constructed by averaging the scores of the corresponding 4 items, respectively.

### 3.3 Effects of explanatory task

To examine the effect of the explanatory task (H2), a regression analysis was also done with the amount of reduction in *Pro-extremism* and *Con-extremism* before and after the task as dependent variables and the amount of reduction in the self-rating indicator of one’s level of understanding as explanatory variables (Table 4). We examined whether the effect of the task on the reduction of extreme attitudes could be explained by the change in reported understanding. For *Pro-extremism*, the amount of reduction in the reported understanding of symptoms and the infection risk was significantly associated with a reduction in this extreme attitude. This suggests that the more the self-rating of one’s understandings of the Covid-19 symptoms and its risk of infection decreased, the more the extreme attitudes in support of self-restraint tended to be moderate. For *Con-extremism*, the amount of reduction in the reported understandings of symptoms and the effects of self-restraint was significantly associated with a reduction in this extreme attitude, indicating that the lower the self-rating of these understandings, the more the extreme attitudes against self-restraint tended to be moderate.

**Table 4:** Results of regression analysis of changes in extreme attitudes associated with changes in self-ratings of understanding.

	Change in *Pro-extremism*		Change in *Con-extremism*	
Explanatory Variables	*β*	t-stat.	*β*	t-stat.
Change in self-ratings of understanding
Symptoms of COVID-19	0.14**	2.37	0.10*	1.65
Infection risks of COVID-19	0.18***	2.92	0.02	0.26
Effects of self-restraint	−0.07	−1.18	0.12**	2.05
Gender	−0.02	−0.35	0.00	0.03
Age	0.04	0.99	0.01	0.13
High school dummy	−0.11	−1.13	−0.07	−0.66
Collage dummy	0.04	0.37	−0.08	−0.84
Nagelkerke *R^2^*	0.07		0.03	

*p < 0.10.

**p < 0.05.

***p < 0.01.

Dependent variables of Pro-and Con-extremism were constructed by averaging the scores of the corresponding 4 items, respectively.

## 4 Discussion

The survey results showed an overall disposition towards extremism in favour of self-restraint among the respondents. As also stated elsewhere in the previous sections, this survey was conducted at a time (March 2021) when COVID-19 was beginning to spread again. So, the results obtained may reflect people’s vigilance about infection as well as their demand for further restrictions. The general trend towards the support for the government’s response to COVID-19 has been observed in European countries as well (Amat et al., 2020; Jørgensen et al., 2021), but it is possible that this trend was particularly strong in Japan, along with the voluntary compliance with self-restraint and its deference to social norm pressures (Borovoy, 2022). Moreover, these trends were found to be particularly strong among women and older people, which is consistent with the existing studies (e.g., Dai et al., 2020; Barber and Kim, 2021; Triberti et al., 2021), which indicate that the woman and elderly people are more likely to comply with the preventive measures against COVID-19.

Both *Pro-and Co-extremism* tend to be associated with a lack of basic knowledge about the symptoms, risks, and characteristics of COVID-19. This finding supports our hypothesis H1 and suggests that extreme attitudes towards self-restraint may not be based on an adequate knowledge of COVID-19. People with *Pro-extremism* tend to lack the knowledge of COVID-19 infection risk and characteristics; they are likely to overestimate the COVID-19 risk and become overly fearful of COVID-19. On the other hand, people with *Con-extremism* lack the knowledge of symptoms and characteristics of COVID-19; they are likely to underestimate the risks of COVID-19 and become overly optimistic about COVID-19. Nevertheless, it was also found that people with extremism on both sides tend to rate their own correct responses to the questions about COVID-19 higher than they actually were. Thus, people’s extreme attitudes for and against self-restraint can be characterized by the lack of knowledge about the virus as well as by the failure to recognize the knowledge lack.

Jørgensen et al. (2021) attributes people’s support for the government’s stringent responses to COVID-19 to their knowledge about the coronavirus and protective behaviors. They found that individual’s self-assessed knowledge about COVID-19 is a key determinant of his or her support for the government’s responses. However, our findings suggest that these self-assessments may lead to overconfidence, as also highlighted by Thaller and Brudermann (2020), in one’s own knowledge of this virus, encouraging not only extremism on the affirmative side of restrictive measures but also extremism on the adverse side. Moreover, proper knowledge of COVID-19 was found to be associated with moderate positions on the restriction measures.

Overall, the extreme attitudes of the respondents did not vary significantly throughout the explanatory task. Also, unlike Rozenblit and Keil (2002), the respondents’ reported understanding varied only slightly in our study. There are two potential reasons for this result. First, Sloman and Vives (2022) show that the experimental effect of explanation is limited for policy issues involving protected values. Their experiment shows that even if self-ratings of understanding are reduced through explanation, attitudinal extremity are not reduced. However, in our experiment, self-ratings of understanding, in addition to the extremism, also did not decrease significantly. The second possible reason is that the respondents may not have been able to recognize their own lack of understanding in the task of explaining the social and economic effects of self-restraint, for which the “correct” answer is not always objectively certain. Therefore, it might be difficult to induce “intellectual humility” (Sloman and Vives, 2022) from the respondents through the explanation, and their ratings of understanding and attitudinal extremity may not have changed significantly.

Thus, the experimental effect, considering all the respondents in our study was limited, but there was a significant association between the changes in extreme attitudes and the changes in reported understanding for both *Pro-extremism* and *Con-extremism*. The results indicate that the individual extreme attitudes could be mitigated if the self-ratings of their own understanding were reduced through the explanation task. In other words, it indicates that those with the extreme opinions rated their own understandings of COVID-19 and the effects of self-restraint highly prior to the explanation task, and thus were likely to suffer from the illusion of understanding, as also discussed by Rozenblit and Keil (2002). Therefore, the more such an illusion of understanding was mitigated through the explanatory task, the more the extreme attitude could have been mitigated. This supports our second hypothesis (H2).

Many studies have noted a tendency for people’s reactions to COVID-19 to polarize (e.g., Dyer, 2020; Hart et al., 2020; Mehrotra, 2020; Kerr et al., 2021), but relevant measures to mitigate extreme attitudes have not been adequately explored. Our study provides some important insights into educational or knowledge-based measures to prevent polarization around the government’s handling of viral infections such as COVID-19. Our findings suggest that the extremism regarding self-restraint may entail a twofold challenge in properly evaluating the effects of such restrictions. First, those with extremism may not have sufficient knowledge of COVID-19. Second, they are nonetheless unaware of their lack of knowledge. They could be perceived as lacking the metacognitive skills necessary for accurate self-assessment (Kruger and Dunning, 1999). Attempts to provide them the knowledge about COVID-19 may not always prove to be successful because they assume that they already have the knowledge. To mitigate their extreme attitudes, the first step may be to provide them opportunities to make them aware of their lack of understanding of COVID-19 through explanatory work on the causal mechanisms by which the restriction policy works, as in this study. Furthermore, as suggested in an existing study on protection values (Baron and Leshner, 2000), it may be useful to ask people to consider the counterexamples that the position they support can have disadvantages for the society (e.g., excessive restrictions can promote social isolation). When health authorities engage in public communication to disseminate knowledge about COVID-19, they could enhance the effectiveness of communication by first providing the recipients with an opportunity of such explanation and reflection.

Nevertheless, several issues remain unaddressed in this study. First, the phenomenon of polarization can be explained by several psychological factors in addition to the illusion of understanding focused on in this study; for example, they are easily influenced by biased media information (Lippmann, 1922), they seek information that endorses their current position (Nickerson, 1998), they process new information in a biased manner that reinforces their initial position (Lord et al., 1979), and they associate with others who have similar preferences (Lazarsfeld and Merton, 1954). Future research should explore these psychological processes underlying the development of extreme attitudes. Second, the explanatory tasks to make the respondents aware of their own lack of understanding regarding COVID-19 were limited in this study. An important issue is to consider practical ways to make them steadily aware of their lack of understanding through additional measures, such as by providing feedback on the validity of their explanations about the causal mechanisms by which the restriction policy works. Third, the respondents in this study were recruited online and may be a sample particularly susceptible to the influence of social media, known to often contain misleading information (Li et al., 2020; Vancini et al., 2021), and thus prone to polarization (Hart et al., 2020). Future investigation with a wider sample should be conducted to confirm the generality of the findings of this study. Fourth, we have not analysed the content of the open-ended explanations provided by the respondents. The details of their descriptions may be related to the experimental effects on the awareness of lack of understanding and the moderation of extremism, which will be the subject of our future research.

## 5 Concluding remarks

The outbreak of COVID-19 has revealed that our society is constantly exposed to the threat of viruses. Because it is almost impossible to completely eliminate the risk of virus infection, our society must ‘get along’ with the risk. Extremism focused on in this study, whether in favor of or against COVID-19 restrictions, is a major sacrifice to people’s health or daily lives and is contrary to a better way of dealing with the virus risk. The results of this study show that not only do the people with extremism lack the knowledge about COVID-19 but they are also less self-aware of their own insufficient knowledge. This finding, however, implies that extreme attitude could be mitigated if people could be aware of their lack of understanding about COVID-19. Thus, our ‘knowledge of ignorance’ in the sense of first knowing our lack of understanding will contribute to the formation of a society resistant to viral risk, based on a better relationship with the virus.

## Data availability statement

The datasets presented in this study can be found in the online repository, https://data.mendeley.com/datasets/6j8gmrr2gx/1.

## Author contributions

TH: Conceptualization, Formal analysis, Investigation, Methodology, Project administration, Writing – original draft, Writing – review & editing. NB: Conceptualization, Project administration, Writing – review & editing.
