# Supplementary material for: Extremism, knowledge, and overconfidence in the covid-19 restriction times
Source: Front Psychol. 2024 Feb 1;15:1295807. doi: 10.3389/fpsyg.2024.1295807 (PMC10867172; doi:10.3389/fpsyg.2024.1295807)
Supplement: Supplementary file 1 [file Table_1.DOCX]

**Supplemental Material: Survey Instruments**

**Before explanation task**

***Pro-extremism***

Question:

Unless there is a zero chance of being infected with the COVID-19, I think we should thoroughly exercise self-restraint.

I think we should give priority to not spreading the infection anyway, no matter how much our normal lives are sacrificed.

No matter how much the Japanese economy worsens, I think we should give priority to not spreading the infection anyway.

I think we should refrain from going out anyway until the infection of the COVID-19 is completely controlled.

Scale: *“Strongly disagree”* (1) — *“Strongly agree”* (7)

***Con-extremism***

Question:

Even if there is a possibility of being infected with the COVID-19, I don't think it is necessary to restrain oneself at all.

Even if there is a possibility of spreading the infection, I think that we should prioritize our normal life as before.

Even if there is a possibility of spreading the infection, I think priority should be given to promoting economic activities in Japan.

Even if there is a possibility of being infected with the COVID-19, I don't think it is necessary to refrain from going out at all.

Scale: *“Strongly disagree”* (1) — *“Strongly agree”* (7)

***Self-rating of understanding***

Question:

I think I understand well the symptoms of the COVID-19.

I think I understand well the risk of infection by the COVID-19.

I think I understand well the effects and impact of restrictions of social and economic activities.

Scale: *“Strongly disagree”* (1) — *“Strongly agree”* (7)

***Questions about symptoms of COVID-19***

Question: Is each of the following a symptom of the new coronavirus?

Choices: *Fever; Damp cough with phlegm; Malaise; Muscle aches; Sore throat; Diarrhea; Headache; Conjunctivitis; Loss of taste or smell; Skin rash; Discoloration of hands, feet, or fingers; Dyspnea or shortness of breath; Chest pain or pressure; Loss of speech or motor function*

Question: Of the 14 questions you answered above, how many do you think you answered correctly?

Answer: (1) — (14)

***Questions about infection risks of COVID-19***

Question:

How likely do you think you are to become infected once you go out?

How likely do you think you are to become infected if you come into contact with an infected person?

How likely do you think you are to become infected if you share food with an infected person?

How likely do you think you are to become infected if you live with an infected family member?

How likely do you think you are to become infected once you use public transportation?

Suppose there is a person in front of you who is infected with the novel coronavirus. The person is not coughing, talking, or standing still. He or she is not wearing a mask. The room is ventilated. What do you think the chances are that you are still infected?

What percentage of infected people with symptoms do you think will become seriously ill?

What percentage of people infected with the novel coronavirus do you think will infect others?

Of those 60 years of age or older who are infected with the novel coronavirus, what percentage do you think will die? (Assume that there is adequate medical care.)

Of those 40 years of age or older who are infected with the novel coronavirus, what do you think the probability is that they will die? (Assume that there is adequate medical care.)

Of those under 40 years of age who are infected with the novel coronavirus, what is the probability that they will die? (Assume that there is adequate medical care.)

Scale: *“0% to less than 2%”* (1); *“2% to less than 5%”* (2); *“5% to less than 10%”* (3); *“10% to less than 20%”* (4); *“20% to less than 30%”* (5); *“30% to less than 40%”* (6); *“40% to less than 50%”* (7); *“50% to less than 60%”* (8); *“60% to less than 70%”* (9); *“70% to less than 80%”* (10); *“80% to less than 90%”* (11); *“90% or more to 100%”* (12)

Question: Of the 11 questions you answered above, how many do you think you answered correctly?

Answer: (1) — (11)

***Questions about characteristics of COVID-19***

Question:

New coronaviruses are transmitted by droplets.

New coronaviruses are transmitted by contact.

New coronaviruses are transmitted by asymptomatic carriers.

New coronaviruses are transmitted by pets.

New coronaviruses are transmitted by flies and mosquitoes.

Infection can occur from letters, imported food, and other packages sent from the place where the infected person was found (foreign or domestic).

New coronaviruses can be transmitted through food.

New coronaviruses are infectious on the surface of objects for up to one week.

Close contacts infected with novel coronaviruses must stay home for 7 days from the day they are identified as close contacts.

Elderly people and people with underlying medical conditions are more likely to have worsening symptoms of novel coronavirus.

Close contacts are those who have had face-to-face contact for more than 5 minutes at a distance (within about 1 meter) that would be within arm's reach if they reached out to each other.

Between novel coronavirus and seasonal influenza, the probability of death is higher with seasonal influenza.

PCR testing is mandatory for any symptomatic infected person as a discharge criterion.

Scale: *“Yes”* (1) — *“No”* (0)

Question: Of the 13 questions you answered above, how many do you think you answered correctly?

Answer: (1) — (13)

**Explanation task**

Task 1) What advantages do you think there would be to self-restraint from social and economic activities? Please describe in as much detail as possible.

Task 2) Which disadvantages do you think there would be in self-restraint from social and economic activities? Please describe in as much detail as possible.

Task 3) What advantages do you think there would be to non-restraint from social and economic activities? Please describe in as much detail as possible.

Task 4) What disadvantages do you think there would be to non-restraint from social and economic activities? Please describe in as much detail as possible.

**After explanation task**

***Pro-extremism***

Question:

Unless there is a zero chance of being infected with the COVID-19, I think we should thoroughly exercise self-restraint.

I think we should give priority to not spreading the infection anyway, no matter how much our normal lives are sacrificed.

No matter how much the Japanese economy worsens, I think we should give priority to not spreading the infection anyway.

I think we should refrain from going out anyway until the infection of the COVID-19 is completely controlled.

Scale: *“Strongly disagree”* (1) — *“Strongly agree”* (7)

***Con-extremism***

Question:

Even if there is a possibility of being infected with the COVID-19, I don't think it is necessary to restrain oneself at all.

Even if there is a possibility of spreading the infection, I think that we should prioritize our normal life as before.

Even if there is a possibility of spreading the infection, I think priority should be given to promoting economic activities in Japan.

Even if there is a possibility of being infected with the COVID-19, I don't think it is necessary to refrain from going out at all.

Scale: *“Strongly disagree”* (1) — *“Strongly agree”* (7)

***Self-rating of understanding***

Question:

I think I understand well the symptoms of the COVID-19.

I think I understand well the risk of infection by the COVID-19.

I think I understand well the effects and impact of restrictions of social and economic activities.

Scale: *“Strongly disagree”* (1) — *“Strongly agree”* (7)
